# Host–commensal interaction promotes health and lifespan in **Caenorhabditis elegans** through the activation of HLH-30/TFEB-mediated autophagy

**DOI:** 10.18632/aging.202885

**Published:** 2021-03-26

**Authors:** Miroslav Dinić, Marija Herholz, Uroš Kačarević, Dušan Radojević, Katarina Novović, Jelena Đokić, Aleksandra Trifunović, Nataša Golić

**Affiliations:** 1 Laboratory for Molecular Microbiology (LMM), Institute of Molecular Genetics and Genetic Engineering (IMGGE), University of Belgrade, Belgrade, Serbia; 2 Cologne Excellence Cluster on Cellular Stress Responses in Ageing-Associated Diseases (CECAD) and Institute for Mitochondrial Diseases and Ageing, Medical Faculty, University of Cologne, Cologne, Germany

**Keywords:** *Caenorhabditis elegans*, autophagy, *Lactobacillus fermentum*, aging, HLH-30

## Abstract

Gut homeostasis is maintained by the close interaction between commensal intestinal microbiota and the host, affecting the most complex physiological processes, such as aging. Some commensal bacteria with the potential to promote healthy aging arise as attractive candidates for the development of pro-longevity probiotics. Here, we showed that heat-inactivated human commensal **Lactobacillus fermentum** BGHV110 (BGHV110) extends the lifespan of **Caenorhabditis elegans** and improves age-related physiological features, including locomotor function and lipid metabolism. Mechanistically, we found that BGHV110 promotes HLH-30/TFEB-dependent autophagy to delay aging, as longevity assurance was completely abolished in the mutant lacking HLH-30, a major autophagy regulator in **C. elegans**. Moreover, we observed that BGHV110 partially decreased the content of lipid droplets in an HLH-30-dependent manner and, at the same time, slightly increased mitochondrial activity. In summary, this study demonstrates that specific factors from commensal bacteria can be used to exploit HLH-30/TFEB-mediated autophagy in order to promote longevity and fitness of the host.

## INTRODUCTION

The gut microbiota has emerged as one of the most important factors that contribute to host health and aging [1]. The interplay between gut microbes and epithelial cells regulates various aspects of gut physiology, including proper epithelial and immune development, immune function and metabolism [2]. High-throughput screening of microbial pro-longevity factors from commensal *Escherichia coli* showed that 29 bacterial genes when deleted, increase the longevity of the host by interacting with several aging-regulatory pathways [3]. Also, the reported age-related changes in the gut microbiota composition, including a decrease in the *Firmicutes* phylum with perturbations in the *Lactobacillus* species diversity, suggest a bacterial impact on host aging [2, 4, 5]. Interestingly, diets based on probiotic lactobacilli supplementation have been shown to improve the state of dysbiosis observed in elderly people [6]. Reports show that beneficial lactobacilli exhibit the potential to stimulate aging-related genes capable of enhancing host longevity and the anti-stress responses [7, 8]. It is important to note that the activation of a specific longevity program is strain-dependent and strongly related to the applied *Lactobacillus* strain, which might interfere with different cellular targets [9].

The roundworm *Caenorhabditis elegans,* with its short and easily monitored lifespan, has been successfully used as a model system in the field of aging research. The worms are reared on a single bacterial strain of *E. coli* and this well-defined microbiota enables using *C. elegans* for studying the relationship between gut microbes and host aging [10]. Several self-protective stress-response regulators, including the forkhead transcription factor FOXO/DAF-16 [11], canonical p38 mitogen-activated protein kinase (MAPK)/PMK-1 [12] and Nrf2/SKN-1 [13], have been found to participate in the longevity assurance in *C. elegans* triggered by different probiotic bacteria.

Interestingly, it has been shown that the surface biomolecules [14] and metabolites [15] of lactobacilli could trigger autophagy, a commonly known pro-survival mechanism responsible for the recycling of long-lived proteins, molecules and organelles [16]. Currently, little is known on whether the activated autophagy could be the mechanism responsible for the pro-longevity effects of lactobacilli. In *C. elegans*, one of the major regulators of many autophagy-related genes is the basic helix-loop-helix 30 (HLH-30), a functional mammalian orthologue of the transcription factor EB (TFEB), exhibiting a key role in lifespan determination [17]. Besides autophagy, HLH-30 links lysosomal lipolysis and autophagy to control fat storage [18] and dictates host defense against infection [19]. By controlling the majority of host cytoprotective responses, the activation of HLH-30/TFEB distinguished itself as an attractive target for beneficial microbes to strengthen the host resistance.

Next to the traditional use of probiotics, the application of non-viable microbial cells, microbial fractions or cell lysates (postbiotics), which can mimic the physiological benefits of probiotics in a more controllable way than using live bacteria, started gaining attention for various purposes [20]. Therefore, we report here that the heat-inactivated human commensal *Lactobacillus fermentum* BGHV110 strain extends the lifespan of *C. elegans* in an HLH-30-dependent manner. We showed that increased transcription of the *hlh-30* gene primarily results in the upregulation of autophagy, contributing to the pro-longevity effect of the BGHV110 postbiotic.

## RESULTS

### Heat-inactivated *Lb. fermentum* BGHV110 extends lifespan in *C. elegans*

**Figure 1:**
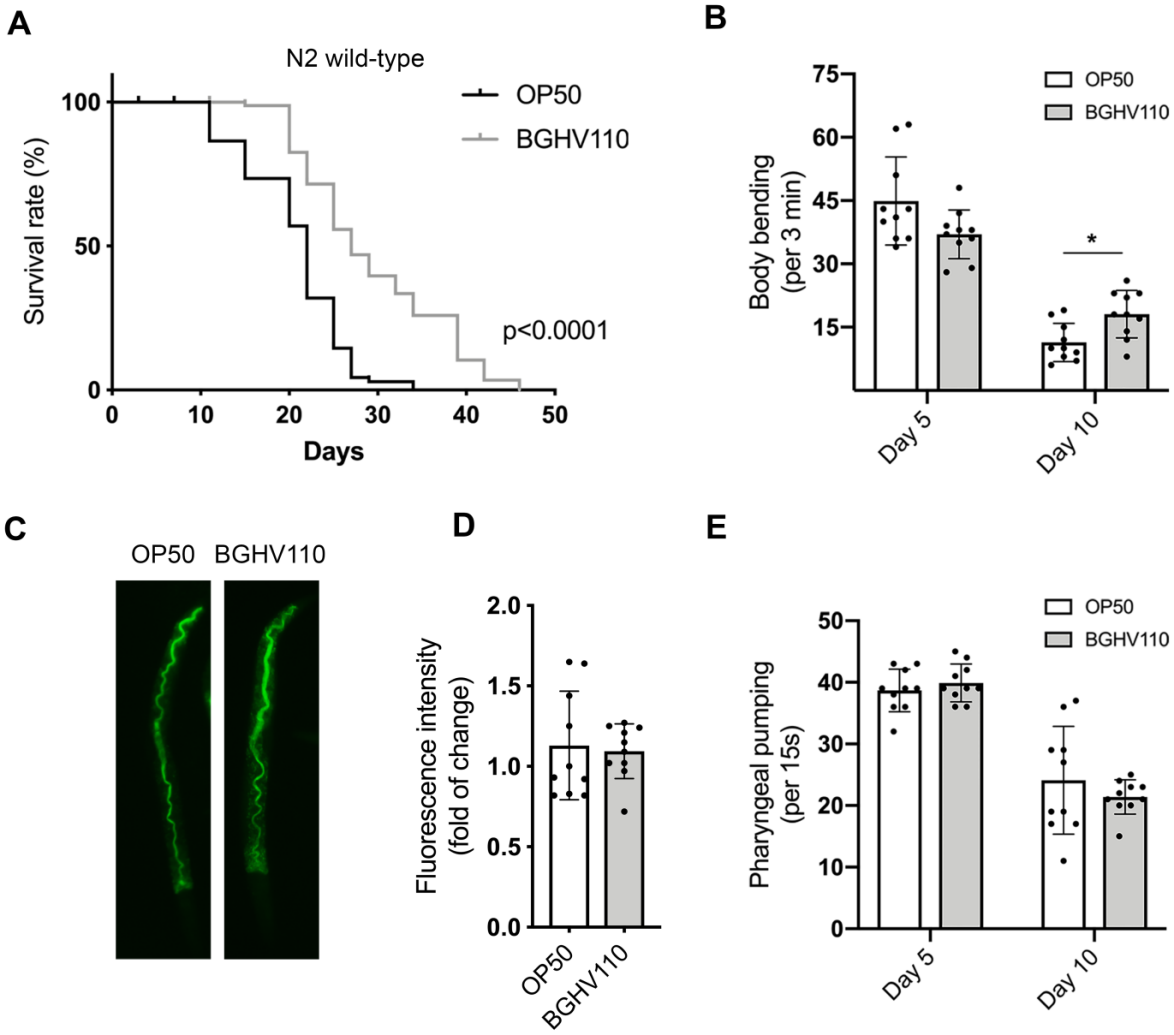
**Heat-inactivated *Lb. fermentum* BGHV110 affects longevity and healthspan in *C. elegans*.** (**A**) Lifespan curve of WT animals fed with heat-inactivated control (OP50) and BGHV110 bacteria from the L4 developmental stage maintained at 20° C (n=100 per group, results from one of two experiments with similar results are shown). (**B**) Body bending rates were measured in WT animals on day 5 and day 10 of adulthood (n=10 per group, results are representative of 3 independent assays). (**C**) Gut localization and (**D**) fluorescence intensity quantification of acridine orange-stained heat-inactivated OP50 and BGHV110 bacteria visualized by fluorescence microscopy in day 1 adult WT animals (n=10 per group, results are representative of 3 independent assays). (**E**) Pharyngeal pumping rates were measured in WT animals on day 5 and day 10 of adulthood (n=10 per group, results are representative of 3 independent assays). All values are presented as mean ± SD. Student’s t-test was used to compare the treated group relative to control (*p < 0.05). The log-rank (Mantel-Cox) test was used to assess the p-value in lifespan analysis.

To investigate the effect of a postbiotic-based diet on organismal aging, we used *C. elegans* as a model system*.* Our previous study identified the prospective postbiotic potential of the strain *Lb. fermentum* BGHV110 in the modulation of autophagy in human hepatoma HepG2 cells [14]. Given the role of autophagy as a longevity-related mechanism [21, 22], we assumed that heat-inactivated BGHV110 could exhibit beneficial effects on the host lifespan. Initially, the effect of the heat-inactivated BGHV110 strain was assessed in a *C. elegans* N2 wild-type (WT) strain. In order to focus our research exclusively on the effect on aging, and not host development, feeding with BGHV110 was started from the first day of adulthood. Hence, age-synchronous worms were grown to the L4 stage on an *E. coli* OP50 strain and then transferred to plates containing heat-inactivated OP50 (control) or BGHV110 bacteria. Lifespan analysis revealed that feeding with BGHV110 significantly extended both median and maximal lifespan in worms compared to the OP50 fed control (Figure 1A).

Since aging also affects different fitness parameters, we next assessed locomotor activity, shown in worms to decline with age. Indeed, in the control animals, we detected a steep decrease in the frequency of body bending with increasing age (Figure 1B). The decline was less prominent in the BGHV110 fed animals that exhibited a higher motility rate on day 10 of adulthood in comparison with the OP50 fed control (Figure 1B). To test if the longevity-promoting signal might be a consequence of the dietary restriction (DR) caused by decreased feeding on the *Lactobacillus* strain, we prestained the BGHV110 with acridine orange and visualized its presence in the worm intestinal lumen. However, no difference in the fluorescence intensity of the bacteria present in the gut lumen was detected between the two groups, suggesting that the increase in lifespan was not caused by the putative DR (Figure 1C, 1D).

We further measured the pharyngeal pumping rate that controls the food intake ability in *C. elegans* and also decreases with age. Although an age-associated decline in the pharyngeal muscle function was detected, no differences in bacterial uptake between the OP50 and the BGHV110 fed animals were observed, either on day 5 or day 10 of adulthood (Figure 1E).

Overall, these results indicate that the heat-inactivated BGHV110 postbiotics are able to delay aging and improve the motility of treated animals independently of the number of ingested bacteria.

### Heat-inactivated *Lb. fermentum* BGHV110 upregulates autophagy

**Figure 2:**
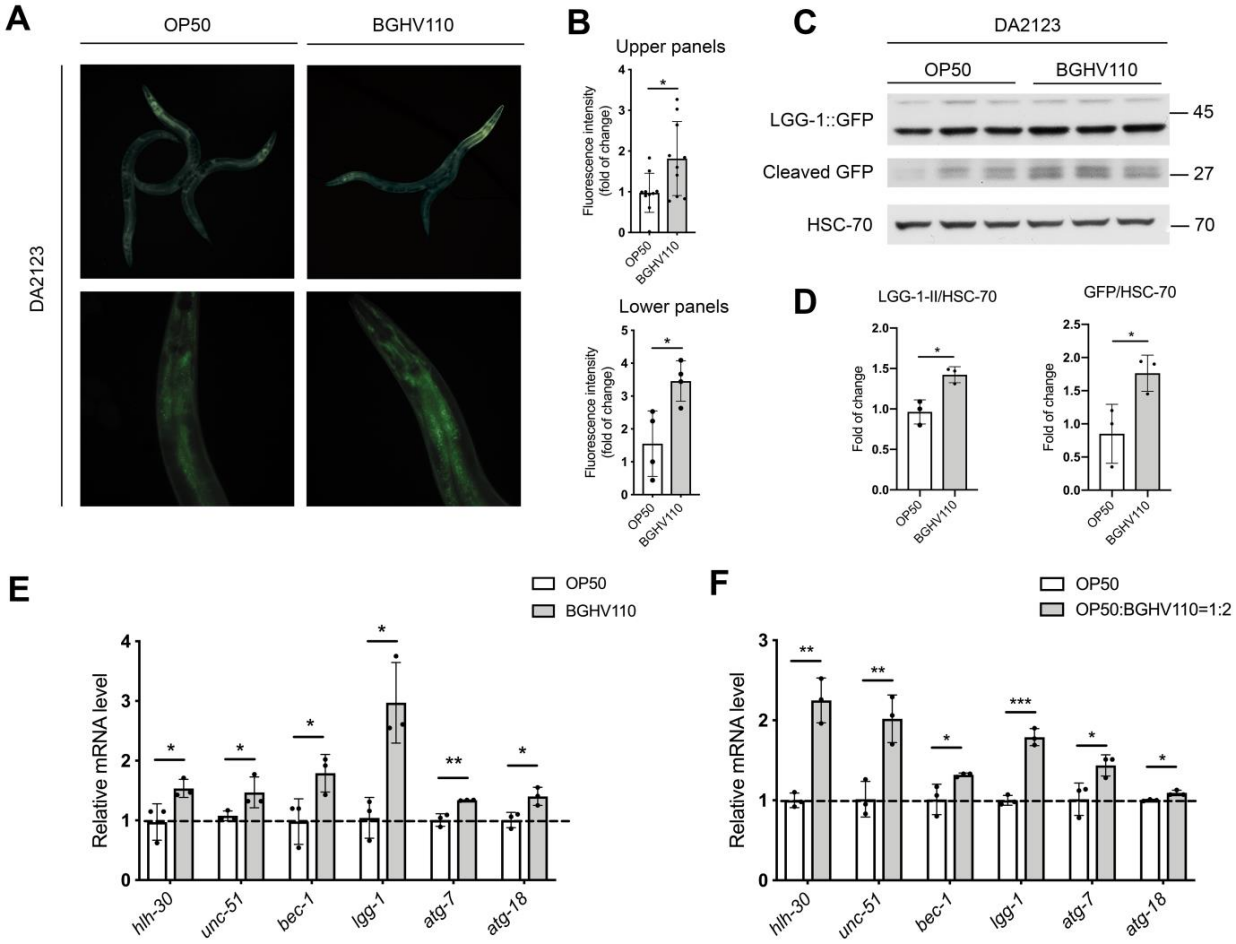
**Heat-inactivated *Lb. fermentum* BGHV110 triggers autophagy in *C. elegans*.** Representative fluorescence images (**A**) and quantifications (**B**) of day 1 adult DA2123 transgenic animals expressing GFP::LGG-1 under the *lgg-1* promoter after overnight BGHV110 treatment (n=4–10, results are representative of 3 independent assays). Western blots (**C**) and densitometric analysis (**D**) showing the levels of GFP::LGG-1 and cleaved GFP proteins isolated from the DA2123 transgenic strain on day 1 of adulthood after overnight BGHV110 treatment. HSC-70 was used as a loading control (n=3, three independent experiments). Expression of *hlh-30* and autophagy-related genes was measured by qRT-PCR in L4 stage WT animals after 6 h of treatment with (**E**) heat-inactivated BGHV110 and (**F**) heat-inactivated OP50 supplemented with heat-inactivated BGHV110 in a 1:2 ratio (n=3, three independent experiments). All values are presented as mean ± SD. Student’s t-test was used to compare the treated group relative to control (*p < 0.05, **p < 0.01, ***p < 0.001).

We next focused on the possible involvement of autophagy in the pro-longevity effect of BGHV110 feeding. We used a reporter DA2123 transgenic strain expressing a GFP-tagged version of LGG-1 under the *lgg-1* promoter, an orthologue of the mammalian LC3 autophagy marker. Our results revealed a significant increase in GFP fluorescence intensity in the worms treated with the BGHV110 strain compared to control (Figure 2A, 2B). To confirm this result, we followed the expression levels of cleaved GFP, which appears upon the formation of the autolysosome, and the LGG-1-II::GFP expression as indicators of autophagy activation by using the Western blot analysis [23]. The intensity of the band corresponding to free GFP was significantly increased in the worms fed with BGHV110, as well as the LGG-1-II/HSC-70 ratio, compared to OP50 control, suggesting an increase in autophagy (Figure 2C, 2D). To gain further insights into the observed activation of autophagy, we analyzed the transcript levels of relevant genes. Using this analysis, we were able to show that the transcript levels of the *unc-51*, *bec-1*, *lgg-1*, *atg-7* and *atg-18,* encoding proteins involved in the different steps of the autophagy process, were all significantly upregulated upon feeding the animals with BGHV110. Interestingly, an increased mRNA level was also observed for the *hlh-30* gene (Figure 2E). However, in order to address the possibility that the activation of autophagy came as a DR-induced phenomenon, we performed an additional experiment where we evaluated the transcript levels of autophagy-related genes after feeding the animals with heat-inactivated OP50 containing heat-inactivated fractions of BGHV110 in a 1:2 ratio. We observed increased transcription of all tested genes in the worms fed with the heat-inactivated OP50 supplemented with BGHV110, which strengthened our conclusion that BGHV110 triggered autophagy independently of the changes in nutrient intake (Figure 2F). Collectively, these results imply that the effect of BGHV110 on the worms’ lifespan could be mediated through the activation of autophagy controlled by HLH-30.

### HLH-30 is necessary for the activation of the longevity program in *C. elegans* triggered by heat-inactivated BGHV110

**Figure 3:**
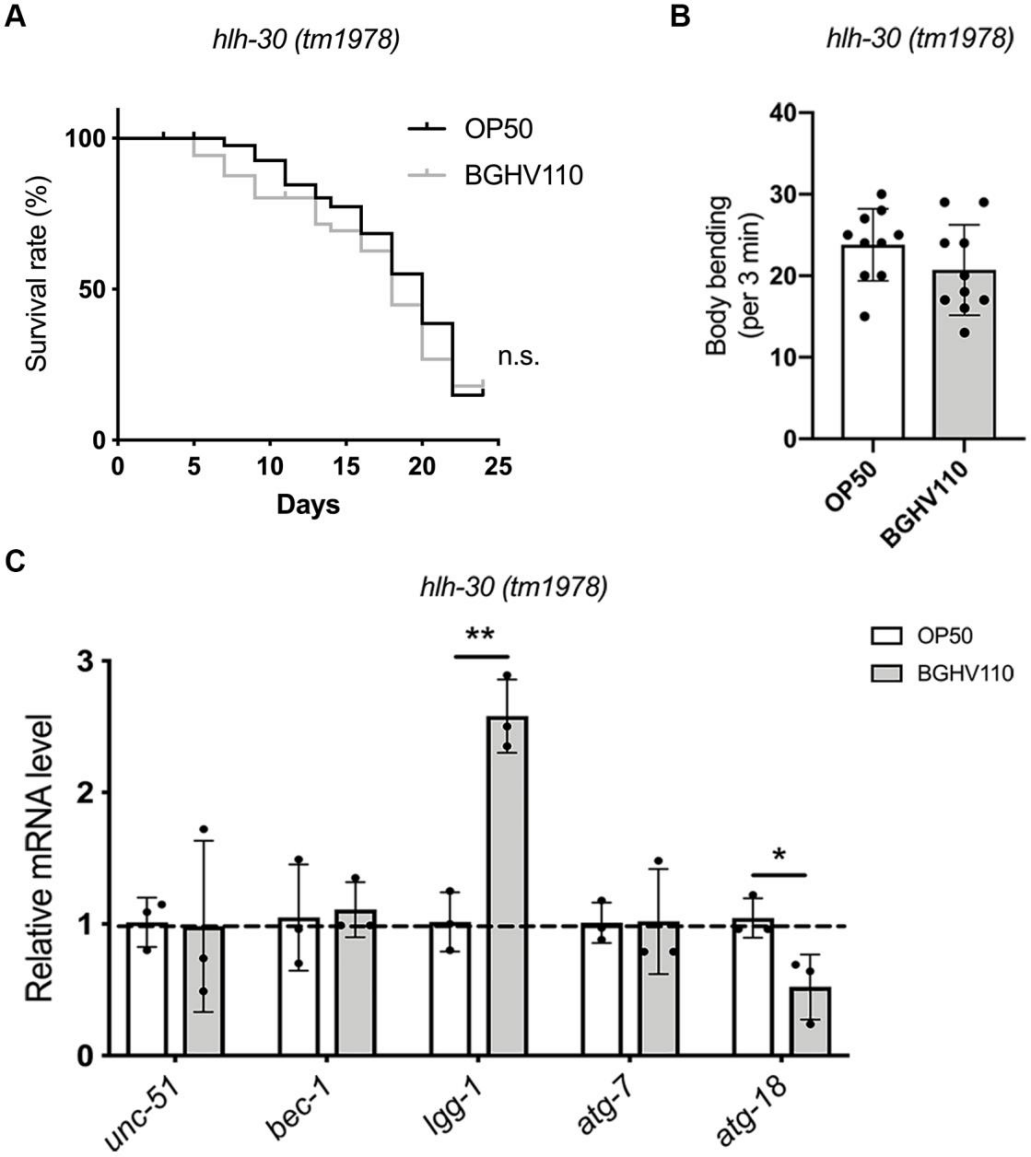
**Heat-inactivated *Lb. fermentum* BGHV110 delays aging in *C. elegans* in an HLH-30-dependent manner.** (**A**) Lifespan curve of the *hlh-30* (*tm1978*) mutant fed with heat-inactivated control (OP50) and BGHV110 bacteria from the L4 developmental stage maintained at 20° C (n=100 per group, results from one of two experiments with similar results are shown). (**B**) Body bending rate was measured in the *hlh-30* (*tm1978*) mutant on day 10 of adulthood (n=10 per group, results are representative of 3 independent assays). (**C**) Expression of autophagy-related genes was measured by qRT-PCR in the L4 stage of the *hlh-30* (*tm1978*) mutant after 6 h of treatment with heat-inactivated BGHV110 (n=3, three independent experiments). All values are presented as mean ± SD. Student’s t-test was used to compare the treated group relative to control (*p < 0.05, **p < 0.01). The log-rank (Mantel-Cox) test was used to assess the p-value in lifespan analysis.

To further elucidate the role of autophagy in the longevity assurance program activated by the BGHV110 postbiotic, we used the *hlh-30* (*tm1978*) *C. elegans* mutant. The lifespan extension caused by BGHV110-feeding in WT worms was completely abrogated in the *hlh-30* mutant (Figure 3A). As expected, feeding of the *hlh-30* mutant with the BGHV110 strain also failed to improve the aging-related decrease of body bending rate at day 10 of adulthood (Figure 3B). To identify what happens with the autophagy markers, which were upregulated in WT animals upon BGHV110 treatment, we performed a qRT-PCR analysis in the *hlh-30* mutant. Notably, most of the genes involved in the autophagy process showed no changes in transcription after the BGHV110 treatment of the *hlh-30* mutant (Figure 3C). Moreover, the transcript level of the *atg-18*, which is required for autophagosome formation and responsible for the longevity phenotype of *C. elegans* [24, 25], was significantly decreased in the *hlh-30* mutant after the BGHV110 treatment (Figure 3C). Surprisingly, feeding with BGHV110 still resulted in an elevated *lgg-1* transcript level in the *hlh-30* animals, demonstrating that a higher *lgg-1* level alone is not sufficient to prolong lifespan. Collectively, these data strongly suggest that BGHV110 activates HLH-30-dependent mechanisms to extend the lifespan of *C. elegans*. These protective responses seem to highly depend on autophagy, as it was not activated in the *hlh-30* mutant.

### Heat-inactivated *Lb. fermentum* BGHV110 reduces lipid droplets

**Figure 4:**
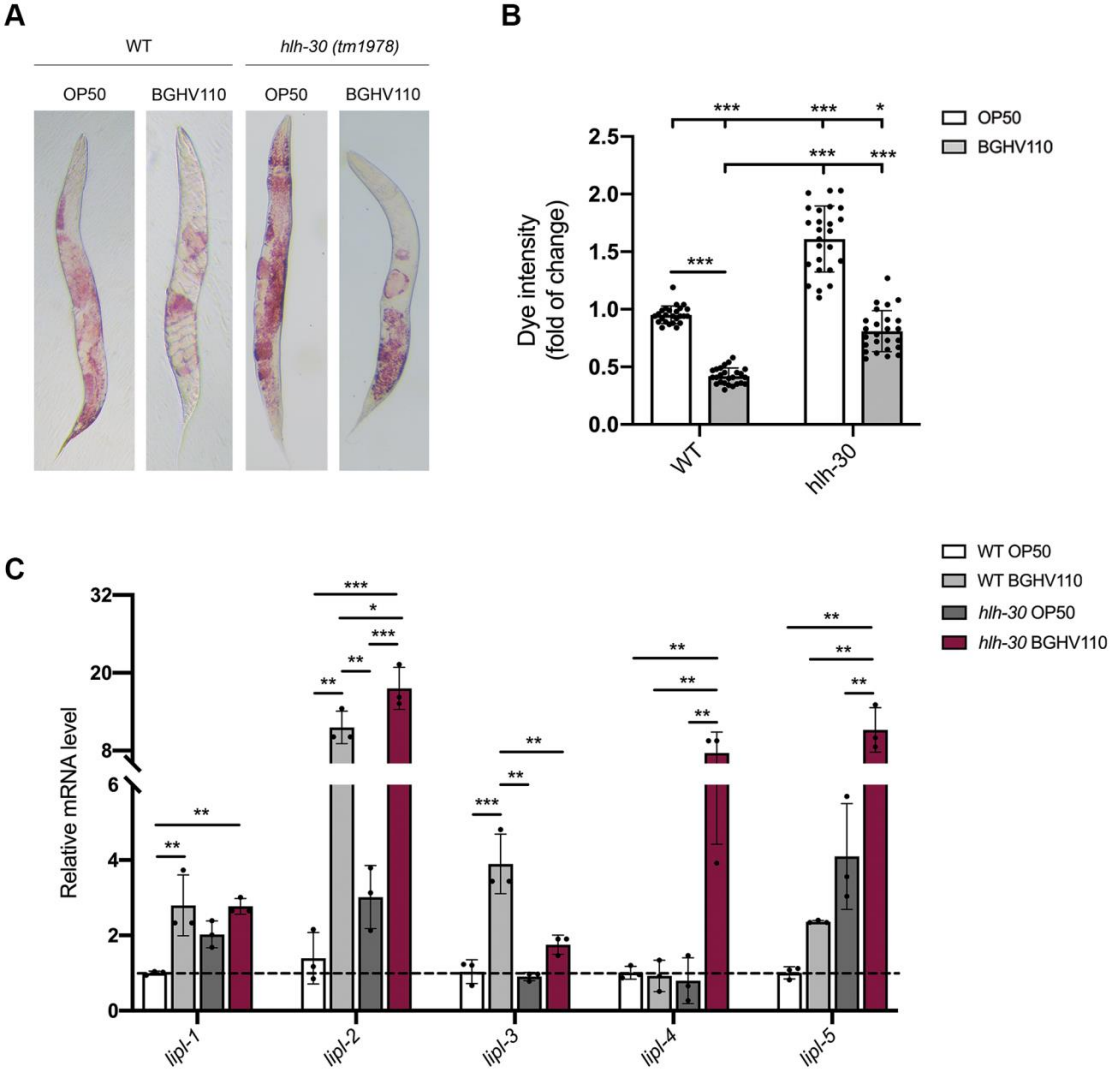
**Heat-inactivated *Lb. fermentum* BGHV110 affects lipid metabolism.** (**A**) Oil Red O staining of the WT and *hlh-30* mutant animals fed with heat-inactivated control (OP50) and BGHV110 bacteria analyzed on day 5 of adulthood. (**B**) Dye intensity quantification of the WT and *hlh-30* mutant animals compared to OP50 control (n=25 per group, results are representative of 3 independent assays). (**C**) The expression of *lipl-1, 2, 3, 4, 5* genes was measured by qRT-PCR in the L4 stage WT and *hlh-30* mutant after 6 h of treatment with heat-inactivated BGHV110 (n=3, three independent experiments). All values are presented as mean ± SD. One-way ANOVA followed by Tukey *post hoc* test for multiple comparisons was used (*p < 0.05, **p < 0.01, ***p < 0.001).

Having in mind that one of the hallmarks of aging is the accumulation of lipids and that HLH-30 could control fat storage in *C. elegans* through a process called lipophagy [18], we analyzed lipid accumulation in WT and the *hlh-30* mutant on day 5 of adulthood. We found that animals fed with BGHV110 showed 2.2-fold lower lipid levels in comparison with the OP50 fed control (Figure 4A, 4B). Surprisingly, we also observed that BGHV110 caused a 2-fold decrease in lipid levels in the *hlh-30* animals compared to OP50 control (Figure 4A, 4B). We further looked into the expression levels of *lipl* genes that encode lipases, the enzymes proposed to break down lipids in *C. elegans* [18]. Upon feeding with BGHV110, elevated transcript levels of the *lipl-1* and *lipl-3* genes were observed in WT animals, exclusively. In contrast, elevated transcripts of the *lipl-4* and *lipl-5* genes were detected only in the *hlh-30* mutant fed with BGHV110. Finally, the BGHV110 diet caused upregulation of the *lipl-2* gene in both *C. elegans* strains (Figure 4C). These results suggest that HLH-30 controls the expression of *lipl-1* and *lipl-3,* which is in accordance with the literature data showing that LIPL-1 and LIPL-3 are the key enzymes breaking down lipids through lipophagy. The LIPL-1 and LIPL-3 are activated by HLH-30 in *C. elegans* [18] and together with LIPL-2 could be responsible for the lipolytic effect induced by BGHV110 mainly through the lipophagy process. Alternatively, activation of different lipases, such as LIPL-4 and LIPL-5, could be related to the reduced lipid content observed in the *hlh-30* mutant treated with BGHV110 in a lipophagy-independent fashion. However, despite the differences in the expression profile of lipases-encoded genes, the reduction of lipid levels in both strains implies that BGHV110 could drive lipid degradation both in an HLH-30-independent and HLH-30-dependent manner. These data indicate that, while the BGHV110 diet controls lipid content in worms, this effect seems to be a consequence of the different *lipl* gene expression profile and not related to the observed increase in longevity.

### Heat-inactivated *Lb. fermentum* BGHV110 does not act through the SKN-1 pathway

**Figure 5:**
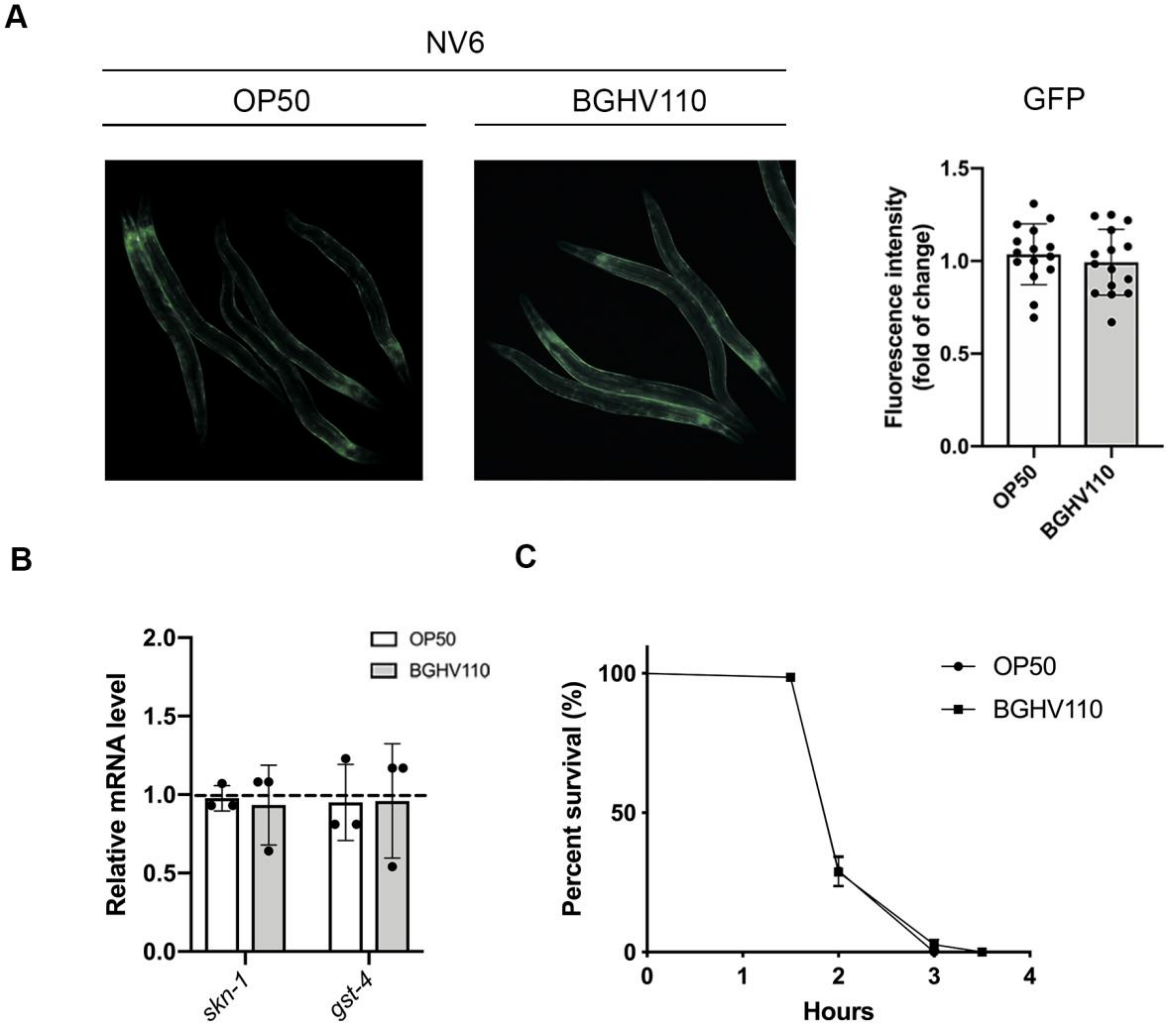
**Heat-inactivated *Lb. fermentum* BGHV110 effects on the SKN-1 detoxification pathway.** GFP fluorescence intensity was visualized by fluorescence microscopy (**A**, left panel) and intensity was quantified (**A**, right panel) in day 1 adult NV6 transgenic animals expressing GFP under the *gst-4* promoter after overnight BGHV110 treatment (n=10, results are representative of 3 independent assays). (**B**) Expression of *skn-1* and *gst-4* genes was measured by qRT-PCR in L4 stage WT animals after 6 h of treatment with BGHV110 (n=3, three independent experiments). (**C**) Overnight OP50 and BGHV110 treated WT animals were exposed to 20 mM H_2_O_2_ on day 1 of adulthood and assayed for survival 4 h later. All values are presented as mean ± SD. Student’s t-test was used to compare the treated group relative to control. The log-rank (Mantel-Cox) test was used to assess the p-value in the H_2_O_2_ assay.

One of the transcription factors that is commonly involved in the regulation of longevity through multiple pathways is SKN-1, mainly acting as a regulator of oxidative stress and xenobiotic responses [13]. We hypothesize that SKN-1 could also be involved in the regulation of the longevity phenotype triggered by inactivated lactobacilli intake. We used a reporter strain harboring GFP expressed under the *gst-4* (glutathione-S-transferases-4) promoter, which is primary regulated by SKN-1. Remarkably, we did not observe any differences in signal intensity between the OP50 and BGHV110 fed animals (Figure 5A). Moreover, the *skn-1* and *gst-4* transcript levels were unchanged upon feeding with BGHV110 (Figure 5B). In agreement with these results, feeding with the BGHV110 strain also failed to ameliorate oxidative stress damage in the functional H_2_O_2_ assay, suggesting that BGHV110 does not influence the general antioxidant activity, nor does it change redox homeostasis to promote lifespan extension in *C. elegans* (Figure 5C).

### Heat-inactivated *Lb. fermentum* BGHV110 slightly affects mitochondrial dynamics without triggering the UPR^mt^ response

**Figure 6:**
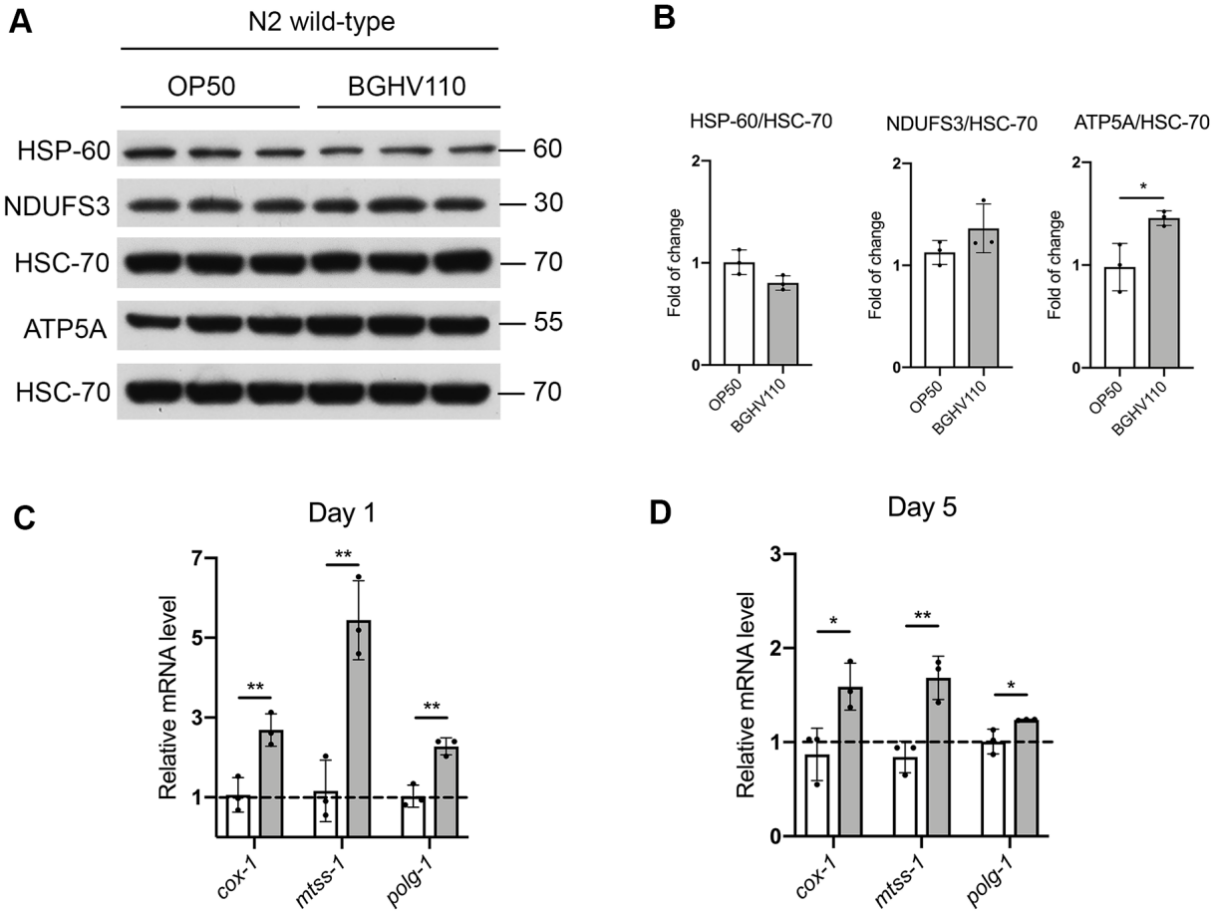
**Mitochondrial homeostasis and the UPR^mt^ assessment.** (**A**) Western blots and (**B**) densitometric analysis showing levels of HSP-60, ATP5A and NDUFS3 proteins isolated from WT animals on day 1 of adulthood after overnight treatment with heat-inactivated BGHV110. HSC-70 was used as a loading control (n=3, three independent experiments). Expression of *cox-1*, *mtss-1* and *polg-1* genes was measured by qRT-PCR after (**C**) 1 day and (**D**) 5 days of treatment with heat-inactivated BGHV110 (n=3, three independent experiments). All values are presented as mean ± SD. Student’s t-test was used to compare the treated group relative to control (*p < 0.05; **p < 0.01).

A recent study showed that five mutants of *E. coli* with different changes in bacterial metabolism increase longevity in *C. elegans*, mainly by regulating mitochondrial dynamics and the mitochondrial unfolded protein response (UPR^mt^) [3]. Therefore, we evaluated the expression levels of heat shock protein 60 (HSP-60), as a commonly used marker of UPR^mt^ activation. However, no differences in HSP-60 expression were observed between the OP50 and the BGHV110 fed worms (Figure 6A, 6B). Assessment of mitochondrial homeostasis was performed by analyzing steady-state levels of NADH dehydrogenase [ubiquinone] iron-sulfur protein 3 (NDUFS3) and ATP synthase subunit 5 (ATP5A), subunits of the mitochondrial respiratory (OXPHOS) complexes I and V, respectively. The obtained results indicated that feeding with BGHV110 increased the amount of the OXPHOS subunits, ATP5A significantly (Figure 6A, 6B). To further address mitochondrial involvement in the health-promoting potential of BGHV110, we analyzed the transcription of the mtDNA encoded subunit I (*cox-1*) and the genes involved in mtDNA replication (*mtss-1* and *polg-1*). We detected elevated transcript levels of all tested genes after 1 day and 5 days of treatment with BGHV110, suggesting higher mitochondrial activity in the worms treated with BGHV110 (Figure 6C, 6D). This result was in accordance with the elevated protein level of complex V, supporting the conclusion that BGHV110 does not activate the UPR^mt^ protective response, but affects mitochondrial homeostasis, most likely through the modulation of mitochondrial biogenesis.

## DISCUSSION

Our data provide strong evidence that feeding with the human commensal *Lb. fermentum* BGHV110 strain, as a postbiotic, extends the lifespan and fitness of *C. elegans*. Further, we identified the HLH-30/TFEB transcription factor as the main regulator of the longevity program initiated by feeding with the BGHV110 commensal bacteria, while disputing the roles of oxidative stress, mitochondrial dysfunction and lipid metabolism in this process.

In the general sense, the probiotic–host cross-talk is reflected in the interaction between bacterial cell surface and secreted macromolecules [26, 27], as well as metabolites [15] that can interact with the host receptors. Indeed, we have previously demonstrated the cytoprotective effect of cell surface macromolecules of the BGHV110 postbiotic in a mammalian cell culture [14]. Here, we focused instead on their potential anti-aging effect on the whole organism by using *C. elegans* as an *in vivo* model. Previous studies have shown that feeding *C. elegans* with heat-, UV- or antibiotic-treated *E. coli* could extend the lifespan by preventing bacterial proliferation and decreasing bacterial metabolism in the gut [28, 29]. This so-called postbiotic concept, which includes replacing live bacteria with dead ones or with defined bacterial metabolites, lowers the risks of high immune stimulating potential. Furthermore, the administration of postbiotics ensures stable biological effects that do not depend on live bacteria or changes in bacterial metabolism [30, 31]. Taking into account that, in this study, postbiotics obtained from heat-inactivated cells of BGHV110 were used, it seems that the observed prolongation of *C. elegans* lifespan could be a consequence of the longevity signals coming from bacterial surface molecules or some heat-resistant microbial metabolites with retained bioactivity. This conclusion is consistent with a recent study showing that feeding with both live and dead *Lactobacillus gasseri* SBT2055 increased the lifespan of *C. elegans,* indicating that the functional molecule is most likely a surface biomolecule or some metabolite with stable bioactivity [13].

HLH-30/TFEB is the master regulator of lysosomal biogenesis that controls the expression of lysosomal hydrolases, membrane proteins and other genes involved in autophagy [32]. The tendency of HLH-30/TFEB to respond to bacterial infection during an innate immune response to *Staphylococcus aureus* infection in *C. elegans* has been previously described [19]. Also, it has been shown that *E. coli* activates TFEB and enhances lysosomal function via TLR4 in mouse macrophages [33], confirming that TFEB could be activated as a result of bacterial–host interaction. Consistently, an interesting hypothesis has proposed that cells use the TFEB pathway as an additional mechanism to activate innate immunity and autophagy in response to bacteria [34]. Our data collected from WT animals and the *hlh-30* mutant indicate that BGHV110 activates HLH-30/TFEB and its downstream targets to promote lifespan and maintain locomotor function in *C. elegans*. Our results pointed to autophagy as a key pathway responsible for the longevity program initiated in *C. elegans* upon feeding with BGHV110. These findings are completely comparable with the upregulation of autophagy and prolonged lifespan in *C. elegans* induced by the anti-aging drug verapamil [35]. Given the crucial role of nutrient deprivation in the induction of autophagy and lifespan determination [36], it was important to distinguish between the upregulation of autophagy due to possible lower intake of BGHV110 and the autophagy promoted by bacterial biomolecules. Although it is very challenging to separate the nutritional from the signaling contribution of BGHV110, the elevated transcript levels of autophagy-related genes induced by the heat-inactivated fractions of BGHV110 and the comparable levels of pharyngeal pumping rate and bacteria intestinal load between control and treatment groups pointed that DR was not responsible for the autophagy induction in *C. elegans*. Moreover, DR-related effects were not observed in the *hlh-30* mutant, which is most likely a consequence of the similar caloric value of *Escherichia* and *Lactobacillus* species [37] and not the lack of nutrients. However, as changes in amino acid levels represent one of the multiple stressors that can activate TFEB [38, 39], it is possible to expect that the difference in amino acid content between *E. coli* and *Lb. fermentum* species could be a contributing factor that stimulated the HLH-30/TFEB activation.

The HLH-30/TFEB complex signaling network might affect lipid metabolism by regulating lipophagy [18, 40]. A recent study monitored lipid accumulation as another feature of aging and showed that lactobacilli could decrease the amount of lipid droplets [41]. This observation is in accordance with our results, showing that feeding with BGHV110 decreases lipid levels, as well as the activation of lipases. However, the changes in lipid metabolism were uncoupled from the increased longevity. We propose that differences in the macronutrients of bacterial strains, as well as the different *lipl* gene expression signature, may be responsible for the difference in fat levels observed in WT and the *hlh-30* mutant animals fed on OP50 and BGHV110 bacterial strains. This is in agreement with the study that showed that feeding on different *E. coli* strains regulates fat storage levels in worms, but does not correlate with changes in lifespan [42].

Although the result regarding the potential of lactobacilli to activate the SKN-1 singling pathway in *C. elegans* is negative, the present study demonstrates that the HLH-30-controlled cytoprotective genes potentially overlap with changes in mitochondrial dynamics, which correlates with the growing body of evidence reporting on the emerging role of TFEB in mitochondrial quality control [43].

Overall, our findings uncover an additional mechanism of the host protective program initiated in *C. elegans* by the heat-inactivated *Lb. fermentum* BGHV110, which is important for the selection of probiotics. In view of this study, exploitation of commensal-induced HLH-30/TFEB-mediated autophagy could be further investigated in order to better understand the potential of using probiotics to modulate HLH-30/TFEB activity and promote host lifespan.

## MATERIALS AND METHODS

### *C. elegans* maintenance and strains

The following strains were used in the study: the wild-type N2 (Bristol), DA2123 adIs2122 [plgg-1::gfp::lgg-1], JIN1375 hlh-30 (tm1978), NV6 [pgst-4::nls-gfp]. All nematodes were maintained at 20° C using standard growing protocols [44]. Age-synchronous worms were grown to the L4 stage on nematode growth medium (NGM) plates seeded with a live *E. coli* OP50 strain followed by transfer to NGM plates containing heat-inactivated OP50, heat-inactivated *Lb. fermentum* BGHV110 or heat-inactivated OP50 supplemented with heat-inactivated *Lb. fermentum* BGHV110 in a 1:2 ratio for different treatments.

### Bacteria preparation and treatment

*Lb. fermentum* BGHV110 strain from the collection of the Laboratory of Molecular Microbiology, Institute of Molecular Genetics and Genetic Engineering, University of Belgrade, was used in this study. The strain was grown overnight at 37° C in deMan-Rogosa-Sharpe (MRS) broth (Sigma-Aldrich) under anaerobic conditions using Anaerocult A (Merck, Darmstadt, Germany). The *E. coli* strain OP50 was cultivated overnight in LB medium at 37° C with shaking/aerobic conditions. For the treatments, BGHV110 cells were pelleted by centrifugation at 5000 × g for 10 min at room temperature and washed twice in phosphate-buffered saline (PBS). The washed BGHV110 cells were resuspended in the same volume of LB medium as OP50 and heat-inactivated at 70° C for 70 min. Heat-inactivation was performed for OP50 under the same conditions. Efficacy of heat inactivation was checked by inoculation of bacterial suspension (10 μl) on MRS/LB agar plates and incubation overnight at 37° C. The heat-inactivated bacterial suspensions, alone or in mixture, were spread on appropriate NGM plates and dried at room temperature.

### Acridine orange staining

Heat-inactivated bacterial suspensions were centrifuged (10000 × g for 5 min) and resuspended in PBS containing acridine orange dye (Sigma-Aldrich) in a final concentration of 10 μM. After 15 min, the suspensions were centrifuged to remove dye and washed three times in PBS. Bacterial pellets were resuspended in LB medium, spread on NGM plates and dried at room temperature. After overnight treatment, fluorescence was examined microscopically.

### Lifespan analysis

For the lifespan analysis, 25 worms per 3.5 cm plate in the L4 developmental stage were transferred to OP50 or BGHV110 NGM plates containing 20 μM of 5-Fluorodeoxyuridine (FudR, Sigma-Aldrich) to avoid progeny hatching. In total, 100 worms (4 plates) were used per condition. First day of adulthood was defined as day 1 in lifespan analysis. Animals were examined every second day by prodding with a silver wire and live worms were transferred to fresh plates. The worms that escaped, or died due to internal hatching or protrusions, were censored.

### Movement

Body bending analysis was performed on day 5 and day 10 of adulthood by transferring worms to non-seeded 9 cm NGM plates. After a short incubation period, the body bending of worms was assessed as the number of full sinusoidal curves the worms made by moving forward or backward. The results are presented as the number of body bends counted over a period of 3 min.

### Pharyngeal pumping assay

Pharyngeal pumping analysis was performed on day 5 and day 10 of adulthood by counting the number of the pharynx rear bulb movements. During the experiment, the animals were placed in bacterial lawns using silver wire. The results are presented as the number of pumping rates in a period of 15 s.

### RNA isolation and quantitative real-time PCR (qRT-PCR)

Total RNA was isolated from approximately 200 worms using a Trizol reagent (Invitrogen). DNase I treatment was performed using an Ambion DNA-free™ Kit (Thermo Fisher Scientific). Reversed transcription was done using 1 μg of isolated RNA as a template, according to the manufacturer′s protocol (Thermo Fisher Scientific). Random hexamers (Applied Biosystems) and RiboLock RNase inhibitor (Thermo Fisher Scientific) were used in the reactions. Synthesized cDNA was further amplified in a 7500 real-time PCR system (Applied Biosystems) using SYBR™ Green PCR Master Mix (Applied Biosystems) under the following conditions: 10 min at 95° C activation, 40 cycles of 15 s at 95° C and 60 s at 60° C. The results were normalized against the *act-1* gene [13] and expressed as relative target abundance using the 2^-ΔΔCt^ method [45]. Primers used in the study are presented in Supplementary Table 1. All primers were purchased from Thermo Fisher Scientific. For each treatment, three independent replicates were used.

### Western blotting

For protein extraction, approximately 200 worms were collected from 9 cm plates using M9 buffer and washed three times to remove the remaining bacteria. Protein isolation was performed as described previously by Herholz et al. [46]. Protein concentration was measured with the Bradford assay (Thermo Fisher Scientific). The extracted proteins (30 μg) were separated on 12% SDS–PAGE and transferred to a 0.2 mm nitrocellulose membrane (GE Healthcare). Western blotting was performed overnight at 4° C with antibodies against: GFP (1:2000, kindly provided by Jan Riemer), HSC-70 (1:2000, Santa Cruz, #sc-7298), HSP-60 (1:2000, BD Transduction Laboratories, #611562), NDUFS3 (1:1000, Mitosciences, MS112) and ATP5A (1:1000, Mitosciences, MS507). The intensity of the bands was quantified using ImageJ (National Institutes of Health) software. For each treatment, three independent replicates were used.

### Microscopy

Worms were immobilized on 2% agarose pads using 5 mM levamisole buffer and observed under an AxioImager Z.1 epifluorescence microscope. The images were collected with a Hammamatsu camera (OrcaR2) and AxioVision software 4.8. Images were analyzed using ImageJ software and normalized to OP50 control.

### Oil-red O staining

For lipid staining, worms were collected from 9 cm plates using M9 buffer after 5 days of treatment. Worms were washed three times with PBS and fixed in MRWB buffer (1% paraformaldehyde, 80 mM KCl, 20 mM NaCl, 7 mM EGTA, 0.5 mM spermidine-HCl, 0.2 mM spermine, 15 mM PIPES, 0.1% 2-mercaptoethanol) for 1 h. Then, the worms were dehydrated in 60% isopropanol for 15 min and stained with filtered Oil Red O solution (5 mg/ml Oil Red O in 60% isopropanol) for 3 h. After the incubation, the dye was removed and the worms were resuspended in PBS containing 0.01% Triton X-100 and visualized using a DM IL LED inverted microscope (Leica). Relative dye intensity was quantified using ImageJ software.

### H_2_O_2_ resistance assay

Following overnight treatments, worms were picked into 96-well plates filled with M9 buffer containing 20 mM H_2_O_2_ in a final concentration. For both conditions, 12 wells of 6 worms were scored for survival every hour. Results are presented as percentage of survival during 4 h of H_2_O_2_ treatment.

### Statistical analysis

All data are presented as mean values ± standard deviation (SD). The differences between control and experimental groups were compared using Student’s t-test. One-way ANOVA followed by Tukey *post hoc* test for multiple comparisons was used in the lipid staining analysis. The differences between survival curves in lifespan and H_2_O_2_ analysis were assessed using the log-rank (Mantel-Cox) test. A p value less than 0.05 was considered statistically significant. The statistical analysis was performed and graphs were prepared using GraphPad Prism 8 software.

## Supplementary Material

Supplementary Table 1
