# Supplementary material for: Host–commensal interaction promotes health and lifespan in Caenorhabditis elegans through the activation of HLH-30/TFEB-mediated autophagy
Source: Aging (Albany NY). 2021 Mar 26;13(6):8040–54. doi: 10.18632/aging.202885 (PMC8034897; doi:10.18632/aging.202885)
Supplement: Supplementary Table 1 [file aging-13-202885-s001.pdf]

SUPPLEMENTARY TABLE

Supplementary Table 1. List of primers used in the study.

Primer name	Primer sequence 5'–3'	Reference
bec-1 forward	TAAACTGTCAGCATCCGTTGA	This work
bec-1 reverse	AGAGCGTCAGAGCAATCATTACA	
lgg-1 forward	AACGCATCCAACTTCGTCCA	This work
lgg-1 reverse	TCCTCGTGATGGTCCTGGTA	
unc-51 forward	GCTTTTTGGAAACCCCCAGC	This work
unc-51 reverse	CAGCGACTTTGTACCTCGTGA	
hlh-30 forward	GTCCTGGCTCCCAAATCAGA	This work
hlh-30 reverse	GATGCGTCTGCTGCATCTTC	
atg-7 forward	ACTCACAGCTGAAGGTTCTCA	This work
atg-7 reverse	CCAGGCGTGCATCTTCAAAT	
atg-18 forward	TTGAATTCCGACGTGGCGTA	This work
atg-18 reverse	GGTAGACGCTTCTGGCTTGT	
skn-1 forward	TTGGCGTGATGATCAACGGA	This work
skn-1 reverse	ACCGAATGGAGATGCTGGTG	
gst-4 forward	GGCAAGAAAATTTGGACTCGC	This work
gst-4 reverse	ACGGGCTGGTTCAACAACTT	
act-1 forward	TGCAGAAGGAAATCACCGCT	This work
act-1 reverse	CGGACTCGTCGTATTCTTGC	
lipl-1 forward	CTCTGAATCGGTTTGCGCTG	This work
lipl-1 reverse	CACGAGTTGCGTTAAGCTGG	
lipl-2 forward	TTGATGGTTGGTACGAGCTG	This work
lipl-2 reverse	TGCTGGATCCTGTGAACTGT	
lipl-3 forward	CGAGCAAAGTGCAGCATTCA	This work
lipl-3 reverse	CAGAAATCCGAATGCGAGGC	
lipl-4 forward	ACTCAAAAAGTGTCGATCTTGAGTT	This work
lipl-4 reverse	ACGATGTAGGAACTTCTCGGC	
lipl-5 forward	TCAGGATGTTGTGGGAAGCC	This work
lipl-5 reverse	ATCATCCGTGGCAACTGTGT	
cox-1 forward	GGTGAACAGTCTACCCACC	This work
cox-1 reverse	GCTAAATCTACTCTACTTCCAGG	
mtss-1 forward	CGATCTCCAAGTCTACCGTC	This work
mtss-1 reverse	GTCATCAACCTCTTGCTTGC	
polg-1 forward	CTGCCTAATACCGTTGCCTTCTT	This work
polg-1 reverse	TTGGAGCCGTCCGGATT	
